# Exploring the Role of MicroRNAs in Progesterone and Estrogen Receptor Expression in Endometriosis

**DOI:** 10.3390/biomedicines12102218

**Published:** 2024-09-28

**Authors:** Jing-Xian Hon, Norhazlina Abdul Wahab, Abdul Kadir Abdul Karim, Norfilza Mohd Mokhtar, Mohd Helmy Mokhtar

**Affiliations:** 1 Department of Physiology, Faculty of Medicine, Universiti Kebangsaan Malaysia, Kuala Lumpur 56000, Malaysia; joanne.honjx@gmail.com (J.-X.H.);; 2 GUT Research Group, Faculty of Medicine, Universiti Kebangsaan Malaysia, Kuala Lumpur 56000, Malaysia; 3 Department of Obstetrics & Gynaecology, Faculty of Medicine, Universiti Kebangsaan Malaysia, Kuala Lumpur 56000, Malaysia

**Keywords:** endometriosis, microRNAs, estrogen receptor, progesterone receptor, progesterone resistance, target genes

## Abstract

**Background/Objectives**: Patients with endometriosis still respond poorly to progestins due to progesterone resistance associated with microRNAs (miRNAs). The aim of this study was to investigate the expression of selected miRNAs, estrogen receptor (ER)α, ERβ, progesterone receptor (PR)-A and PR-B and to determine the target genes of upregulated miRNAs in endometriosis. **Methods**: In this study, 18 controls, 18 eutopic and 18 ectopic samples were analysed. Profiling and validation of miRNAs associated with functions of endometriosis were performed using next-generation sequencing (NGS) and qRT-PCR. At the same time, the expression of ERα, ERβ, PR-A and PR-B was also determined using qRT-PCR. Target prediction was also performed for miR-199a-3p, miR-1-3p and miR-125b-5p using StarBase. **Results**: In this study, NGS identified seven significantly differentially expressed miRNAs, of which six miRNAs related to the role of endometriosis were selected for validation by qRT-PCR. The expression of miR-199a-3p, miR-1-3p, miR-146a-5p and miR-125b-5p was upregulated in the ectopic group compared to the eutopic group. Meanwhile, ERα and ERβ were significantly differentially expressed in endometriosis compared to the control group. However, the expressions of PR-A and PR-B showed no significant differences between the groups. The predicted target genes for miR-199a-3p, miR-1-3p and miR-125b-5p are SCD, TAOK1, DDIT4, LASP1, CDK6, TAGLN2, G6PD and ELOVL6. **Conclusions**: Our findings demonstrated that the expressions of ERα and ERβ might be regulated by miRNAs contributing to progesterone resistance, whereas the binding of miRNAs to target genes could also contribute to the pathogenesis of endometriosis. Therefore, miRNAs could be used as potential biomarkers and for targeted therapy in patients with endometriosis.

## 1. Introduction

Endometriosis is a common gynaecological disease that affects 6–10% of women in their reproductive age. It is an oestrogen-dependent disease characterised by the growth of endometrial glands and stroma outside the uterine cavity [1]. The prevalence of endometriosis is highest in the ovaries, followed by uterosacral ligaments, ovarian fossa, pouch of Douglas and bladder [2]. Patients with endometriosis experience pelvic pain, dysmenorrhoea, dyspareunia and infertility and have a poor quality of life [3]. Although the pathogenesis of endometriosis is not fully understood, the most widely accepted theory is retrograde menstruation, proposed by Sampson in 1927, in which endometrial tissue migrates through the fallopian tubes into the pelvic cavity [4,5,6]. Other mechanisms could include coelomic metaplasia, Mullerian remnants, bone marrow-derived stem cells, genetics and epigenetics [6,7]. Although progesterone therapy provides temporary relief of pelvic pain, patients still respond poorly to progestin treatments due to progesterone resistance in endometriosis [8,9,10]. 

High estrogen and low progesterone levels are important features of endometriosis. It has been shown that aberrant expression of the estrogen receptor (ER) and the progesterone receptor (PR) plays an important role in the pathogenesis of endometriosis [11]. The expression of ERβ is increased in endometriotic tissue compared to normal endometrium, which suppresses ERα and leads to a high ERβ/ERα ratio. In addition, a lower ERα/ERβ ratio may inhibit PR expression, particularly PR-B in endometriotic stromal cells, which could lead to progesterone resistance in patients with endometriosis [12,13]. Although ectopic tissue shows reduced PR expression, the differences in PR expression in eutopic endometriums between patients with and without endometriosis show contradictory results [14]. Therefore, the mechanisms behind progesterone resistance in ectopic and eutopic endometrial tissue are still unclear [14,15].

MicroRNAs (miRNAs) have been associated with the regulation of progesterone resistance, inflammation, proliferation, angiogenesis, and tissue remodelling in endometriosis [16,17]. They are short, non-coding RNA molecules of 21 to 25 nucleotides in length that regulate gene expression by binding to 3’UTR mRNA and causing either degradation of the mRNA or suppression of translation [18,19]. MiRNAs have been shown to play a negative feedback role in cellular differentiation, proliferation, and migration. Their dysregulation has been linked to various diseases, including cancer, heart failure, and haematopoietic disorders [20]. To date, the miRNA database (miRBase) includes 2812 mature human miRNAs [21]. Previous studies have shown that miRNAs can bind to mRNAs that cause progesterone resistance in endometriosis [22,23,24,25]. In a study in rats with endometriosis, the relationship between miRNAs, ER and PR expression was found to show infertility during the window of implantation [26]. Studies on miRNAs have been conducted to understand the mechanisms of progesterone resistance in endometriosis [27]. Specific miRNAs that could be used as potential biomarkers and for targeted therapy have also been identified in endometriosis such as miR-9, miR-34 and miR-297 [28,29,30].

We hypothesise that miRNA expressions differ between ectopic endometriosis tissue and eutopic endometriums in patients with and without endometriosis. Moreover, miRNAs may regulate the expression of ER and PR in endometriotic tissue. Therefore, the aim of the present study was to investigate the regulation of ERα, ERβ, PR-A and PR-B by miRNAs in patients with endometriosis. Next-generation sequencing (NGS) was performed to profile the expression of miRNAs, and the selected miRNAs associated with endometriosis were validated with qRT-PCR. In addition, ER and PR expression measured by qRT-PCR were correlated with the upregulated miRNAs. Target prediction was also performed using cross-linking and immunoprecipitation (CLIP-Seq) and Degradome-Seq data from the StarBase and TargetScan databases. These findings may improve our understanding of the role of miRNAs in endometriosis and thus enable targeted therapy to improve women’s quality of life.

## 2. Materials and Methods

### 2.1. Clinical Samples

All samples were collected with informed consent from 54 women aged 18 to 45 years who underwent surgery for endometriosis or other benign gynaecological conditions between October 2016 and March 2022. Exclusion criteria included patients with malignancies, peritoneal cavity infections, pregnancy, hormonal therapy, use of antibiotics within the last 7 days, and use of GnRH analogues in the last 3 previous menstrual cycles. The sample size of the groups was calculated using the Fleiss method without and with a correction factor [31]. A total of 18 normal endometrial biopsies without endometriosis (control group) and 18 eutopic endometrial biopsies from laparoscopically confirmed endometriosis (eutopic group) were obtained using a pipelle (MedGyn, Lombard, IL, USA), while 18 women with endometriosis underwent laparoscopic surgical removal of ectopic tissue in the ovaries (ectopic group). The control group was laparoscopically confirmed to be free of disease and without evidence of endometriosis and histologically confirmed to have a normal endometrium. Samples were collected in RNAlater (Invitrogen, Waltham, MA, USA) and stored in the −80 °C freezer. Six controls, seven eutopic and two ectopic samples were sent for small RNA sequencing, while an independent set of samples (10 controls, 10 eutopic, and 12 ectopic samples) were validated by qRT-PCR. All control, eutopic and ectopic samples were used for qRT-PCR analysis of ER and PR expressions. This study was approved by the Research Ethics Committee of the National University of Malaysia (UKM PPI/111/8/JEP-2022-709). 

### 2.2. Hematoxylin and Eosin (H&E) Staining

A small piece of endometrium or endometriotic tissue was fixed in 10% formalin and embedded in paraffin. The tissue was cut into sections of 5 μm and placed on a microscopic slide. It was deparaffinised in xylene, 100% alcohol, 80% alcohol, 70% alcohol and washed with water. The tissue was then stained with haematoxylin and eosin and washed with water. The slide was then dehydrated with 80% alcohol, 90% alcohol, 100% alcohol and xylene. Finally, the slide was dried and the stained tissue section was examined under an inverted microscope by a pathologist to confirm the presence of endometrial glands and stroma in the control and endometriosis samples.

### 2.3. RNA Extraction

Total RNA was extracted from endometrial and endometriotic biopsy tissues using the miRNeasy Mini Kit (Qiagen, Hilden, Germany) according to the manufacturer’s protocol and stored at −80 °C until further analysis. RNA concentration and purity were determined using a Nanodrop spectrophotometer (Denovix, Wilmington, DE, USA) with an acceptable A260/280 ratio of 1.9-2.1. RNA integrity was assessed using the Agilent 2100 Bioanalyzer (Agilent Technologies, Santa Clara, CA, USA) with an acceptable RNA integrity number (RIN) of 7 and above. 

### 2.4. MiRNA Expression Profiling Using Next-Generation Sequencing (NGS)

The cDNA libraries were generated and sequenced using the DNBseq platform (BGI, Tai Po, Hong Kong, China) with a single-end read length of 50 bp and sequencing depth of 28.8 million reads per sample. The raw data with low-quality reads, reads with adaptor sequences, a high number of N bases and read lengths of less than 18 bp were filtered off. The total number of reads is listed in Supplementary Table S1. After filtering, the remaining tags were stored in FASTQ format. The clean reads were aligned to the Homo sapiens reference genome (GRCh38.p13) using Bowtie2 as shown in Supplementary Table S2. The miRBase was used to identify known miRNAs, while miRDeep2 was used to predict novel miRNAs. The classification of small RNAs with the number of reads aligned to miRNAs is shown in Supplementary Table S3. DESeq2 was then used to identify differentially expressed miRNAs based on the normalised read counts, and principal component analysis (PCA) was performed to determine sample quality control. The miRNA gene expression levels are shown in Supplementary Table S4. Differentially expressed miRNAs were classified as significant if they had a log_2_ fold change (FC) ≤ −1 or ≥1 and an adjusted *p*-value of <0.05 using a volcano plot. The *p*-value was subjected to multiple hypothesis testing using the Benjamini–Hochberg method to reduce the false discovery rate. Finally, heatmaps and hierarchical clustering analyses were performed using pheatmap in R package (version 1.0.12). To identify miRNAs associated with endometriosis, the differentially expressed miRNAs were selected from the volcano plot and the heatmap. Subsequently, the literature on miRNAs in endometriosis was searched in the PubMed database (https://pubmed.ncbi.nlm.nih.gov/, accessed on 3 June 2022), and the selected miRNAs were validated by qRT-PCR.

### 2.5. Validation of Selected miRNAs and mRNAs by Quantitative Real-Time Polymerase Chain Reaction (qRT-PCR)

The differentially expressed miRNAs related to endometriosis were selected from the volcano plot and heatmap of NGS data for validation by qRT-PCR. For miRNAs, total RNA was reverse transcribed into cDNA using the All-in-One miRNA First-Stand cDNA Synthesis Kit (GeneCopoeia, Rockville, MD, USA). For mRNAs, total RNA was reverse transcribed into cDNA using the qPCRBIO cDNA Synthesis Kit (PCR Biosystems, London, UK). The expression of miRNAs were determined using All-in-One miRNA assays (GeneCopoeia, Rockville, MD, USA): hsa-miR-199a-3p (HmiRQP0289), hsa-miR-196a-5p (HmiRQP0284), hsa-miR-144-3p (HmiRQP0190), hsa-miR-1-3p (HmiRQP0044), hsa-miR-146a-5p (HmiRQP0196), hsa-miR-125b-5p (HmiRQP0096), the internal control, RNU6-2 (HmiRQP9001) and the universal miRNA adaptor PCR primer (QP029). The QuantiTect Primer Assay and QuantiNova LNA PCR Assay (Qiagen, Hilden, Germany) were used for ERα (QT00044492), ERβ (QT01149953) and the internal control and GAPDH (SBH1220545-200) mRNA primers. The following PR-A and PR-B mRNA primers were purchased from Macrogen, Seoul, South Korea: PR-A, 5′-GACGACGCG TACCCTCTCTA-3′ (forward) and 5′-GTA CAGGATGCACTCCAGGG-3′ (reverse); PR-B, 5′-TGCTGGACAGTGTCTTGGAC-3′ (forward) and 5′-CGGAGCTGTCTCCAACCTT-3′ (reverse). 

qRT-PCR was performed with the CFX96 Real-Time PCR System (Bio-Rad, Hercules, CA, USA) using a qPCRBIO SyGreen Blue Mix (PCR Biosystems, London, UK). The reaction mixture consists of 5 μL of 2× qPCRBIO SyGreen Blue Mix, 0.2 μM of forward and reverse primers, 2 μL of distilled water and 1 μL of cDNA to obtain a reaction volume of 10 μL. The thermal cycling conditions are polymerase activation at 95 °C for 2 min, then 40 cycles of denaturation at 95 °C for 5 s and annealing/extension at 60 °C for 30 s. Melting profile analysis was performed to identify single melting peaks for further analysis. Each sample was performed in triplicate. The miRNA levels were normalised to RNU6-2, while the mRNA levels were normalised to GAPDH. The relative expressions were calculated using the 2^−ΔΔCt^ method.

### 2.6. Statistical Analysis

Statistical analysis of qRT-PCR results was performed using GraphPad Prism 9.5.1 (GraphPad Software, La Jolla, CA, USA). The Shapiro–Wilk normality test was used to check the normality of the data. For normally distributed data, the differences between the control, eutopic and ectopic endometriosis groups were compared using one-way ANOVA and Tukey’s multiple comparison test. For non-normally distributed data, differences between the three groups were compared using Kruskal–Wallis and Dunn’s multiple comparison tests. Data in triplicate are presented as mean ± standard deviation (S.D.), and a *p*-value of less than 0.05 was considered statistically significant.

### 2.7. Target Predictions Using CLIP-Seq and Degradome-Seq Data

The target genes of three upregulated miRNAs (hsa-miR-199a-3p, hsa-miR-1-3p, and hsa-miR-125b-5p) were predicted by StarBase (https://rnasysu.com/encori/, accessed on 13 July 2024). StarBase is a database consisting of miRNA–mRNA interaction maps generated from CLIP-Seq and Degradome-Seq data that identify the binding site of Argonaute protein on mRNAs and the cleavage sites on miRNAs. The expression levels of miRNAs and target genes were then supported by the literature in the PubMed database (https://pubmed.ncbi.nlm.nih.gov/, accessed on 14 July 2024). In addition, the probability of preferentially conserved targeting (P_CT_) between miRNA and target gene was determined using TargetScan v8.0 (https://www.targetscan.org/vert_80/, accessed on 13 July 2024). The P_CT_ of 0.7 and above will be selected as target genes for the miRNAs.

## 3. Results

### 3.1. Patient Demographic Data 

The data on the patients of the collected samples are listed in Table 1. Only the clinical characteristic of dysmenorrhoea showed a significant difference between the ectopic and control groups (*p* = 0.024), while age, race, parity, body mass index (BMI), infertility, dyspareunia and pelvic pain showed no significant differences between the three groups.

### 3.2. Histopathological Examination

Microscopic examination of the normal endometrium, the eutopic endometrium and the ectopic endometriotic tissue from ovarian cysts revealed structures of both endometrial glands and stroma (Supplementary Figure S1) and confirmed the diagnosis of the control and endometriosis groups.

### 3.3. Differentially Expressed miRNAs in Ectopic versus Control and Ectopic versus Eutopic Groups

**Figure 1:**
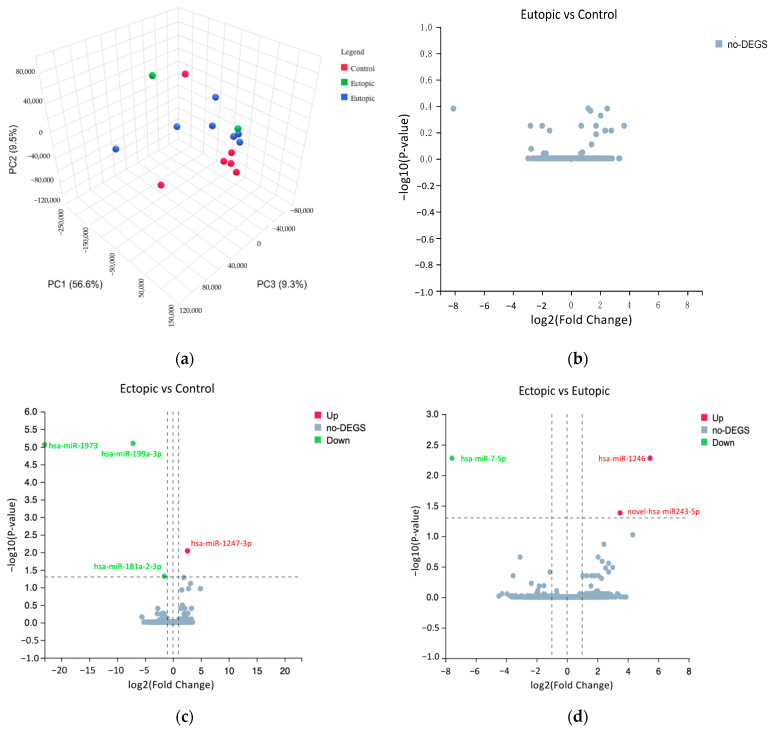

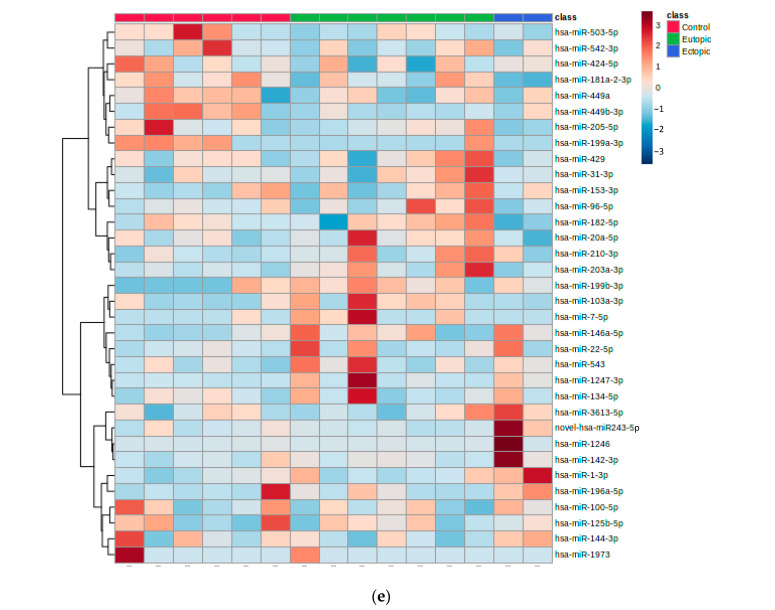
MiRNA expression profiling of endometriosis and control samples. (**a**) The PCA plot showed the grouping of samples into ectopic (green), eutopic (blue) and control (red). Volcano plots of differentially expressed miRNAs showing the relationship between log_2_ (fold change) and −log_10_ (*p*-value) for (**b**) eutopic versus control, (**c**) ectopic versus control and (**d**) ectopic versus eutopic groups. The significantly upregulated miRNAs are shown in red, and significantly downregulated miRNAs are shown in green. (**e**) Heatmap of 34 differentially expressed miRNAs. Samples along the vertical axis are clustered by colour bars. Red indicates the control group, green indicates the eutopic group, and blue indicates the ectopic group. The colour key represents expressions of miRNAs across all samples. Red illustrates upregulation, blue illustrates downregulation, and white shows no changes in miRNA expressions.

NGS was performed on six control, seven eutopic and two ectopic samples, and a total of 2168 miRNAs were detected. When comparing the ectopic and control groups, hsa-miR-1247-3p was significantly upregulated, while hsa-miR-1973, hsa-miR-199a-3p and hsa-miR-181a-2-3p were significantly downregulated (Table 2). In addition, hsa-miR-1246 and the novel hsa-miR-243-5p were significantly upregulated, while hsa-miR-7-5p was significantly downregulated in ectopic versus eutopic samples (Table 3). However, no significant differences in miRNAs were detected between eutopic and control groups. The PCA plot showed a clear separation of the control and eutopic groups, but not in ectopic samples (Figure 1a). The volcano plots showed the relationship between the −log_10_ *p*-value and the log_2_ fold change in the eutopic versus control (Figure 1b), ectopic versus control (Figure 1c) and ectopic versus eutopic (Figure 1d) groups. Hierarchical clustering with a heatmap revealed different expression profiles between ectopic, eutopic and control groups (Figure 1e). 

### 3.4. Validation of Selected miRNAs by qRT-PCR

**Figure 2:**
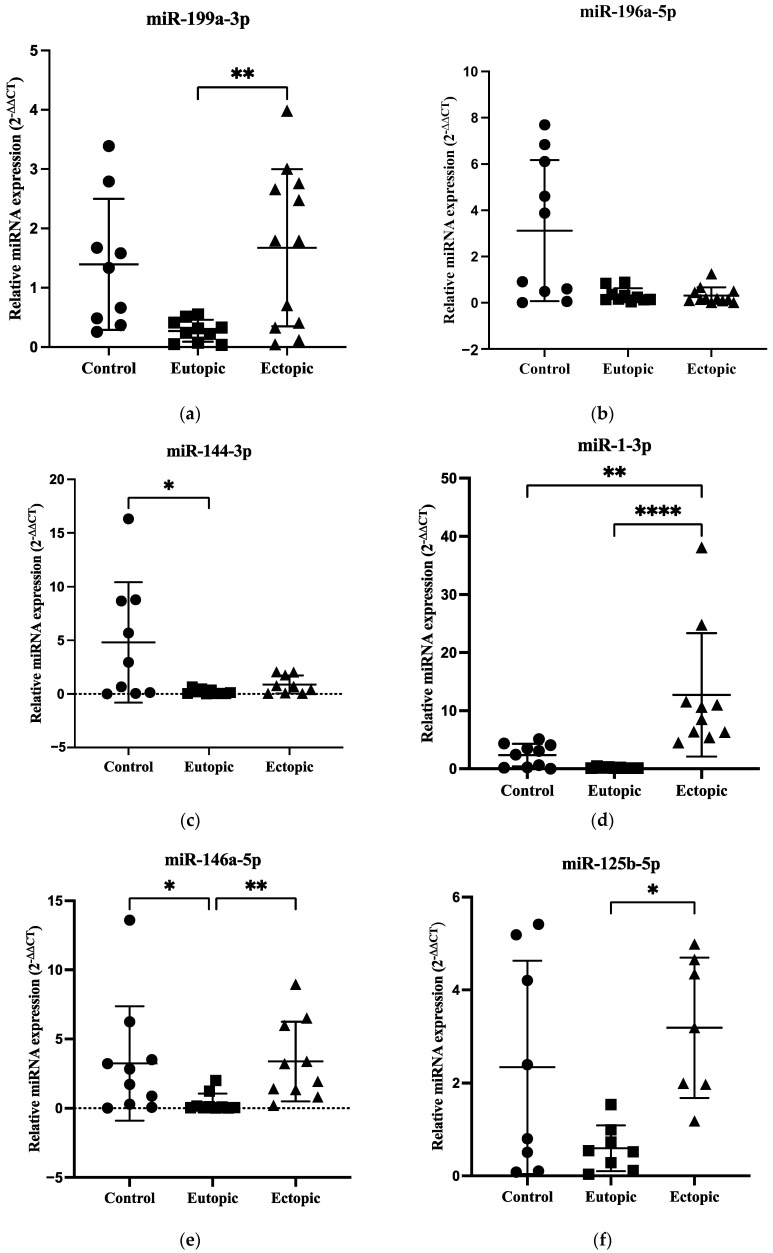
qRT-PCR validation of (**a**) miR-199a-3p, (**b**) miR-196a-5p, (**c**) miR-144-3p, (**d**) miR-1-3p, (**e**) miR-146a-5p and (**f**) miR-125b-5p from NGS data. Data are shown as mean ± SD. * *p* < 0.05, ** *p* < 0.01 and **** *p* < 0.0001. Statistics used were ANOVA or Kruskal–Wallis with Tukey’s or Dunn’s multiple comparisons tests.

**Figure 3:**
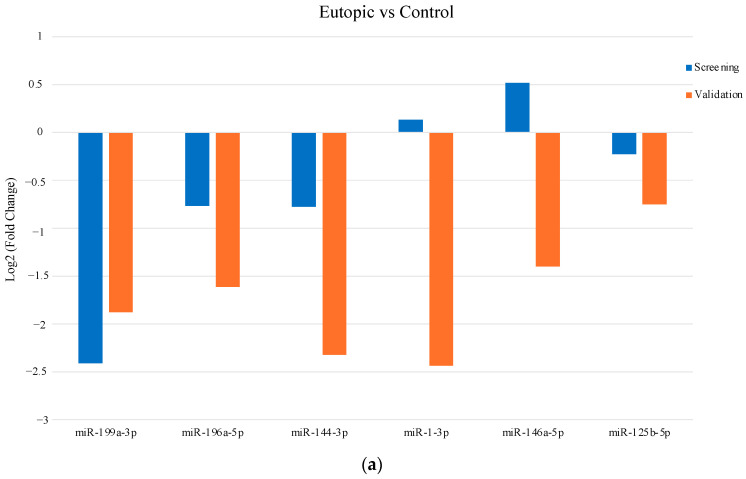

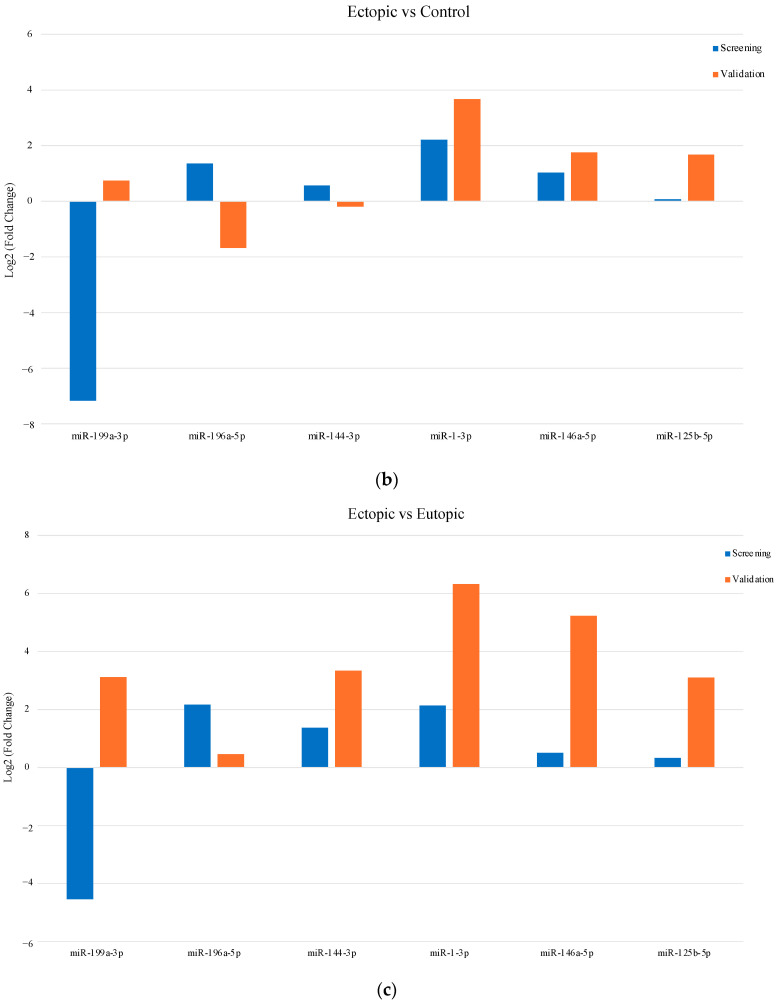
Comparison of NGS and qRT-PCR validation results in the selected miRNAs. The log_2_ fold change of (**a**) eutopic versus control, (**b**) ectopic versus control and (**c**) ectopic versus eutopic groups are shown in screening and validation data.

An independent set of samples (control *n* = 10, eutopic *n* = 10 and ectopic *n* = 12) was subjected to validation by qRT-PCR. Six miRNAs (miR-199a-3p, miR-196a-5p, miR-144-3p, miR-1-3p, miR-146a-5p and miR-125b-5p) related to the roles of endometriosis were selected from volcano plot and heatmap of NGS data for validation. The expression of miR-199a-3p was significantly upregulated in the ectopic group compared to the eutopic group in endometriosis (*p* = 0.0095) (Figure 2a). In addition, there were no significant differences in miR-196a-5p expression between the three groups (Figure 2b), while the expression of miR-144-3p was significantly decreased in eutopic tissues compared to the control group (*p* = 0.0442) (Figure 2c). The expression of miR-1-3p was significantly upregulated in the ectopic group compared to the eutopic and control groups (*p* < 0.0001 and *p* = 0.0098, respectively) (Figure 2d). In addition, the expression of miR-146a-5p was significantly upregulated in the ectopic group compared to the eutopic group in endometriosis patients (*p* = 0.0047), whereas it was significantly downregulated in the eutopic group compared to the controls (*p* = 0.0398) (Figure 2e). The expression of miR-125b-5p was significantly upregulated in the ectopic compared with the eutopic group (*p* = 0.0149) (Figure 2f). The raw data for validated miRNAs is shown in Supplementary Table S5. When comparing the eutopic and control groups, only the validation of miR-199a-3p showed good agreement with the result of miRNA profiling (Figure 3a). In addition, the validations of miR-1-3p and miR-146a-5p agreed with the screening results in the ectopic versus control groups (Figure 3b). In the ectopic versus eutopic samples, the validations of miR-144-3p and miR-1-3p were consistent with the NGS results (Figure 3c).

### 3.5. Expression of ERα Was Decreased with Overexpression of ERβ in Endometriosis, but PR-A and PR-B Showed No Significant Differences between the Groups

**Figure 4:**
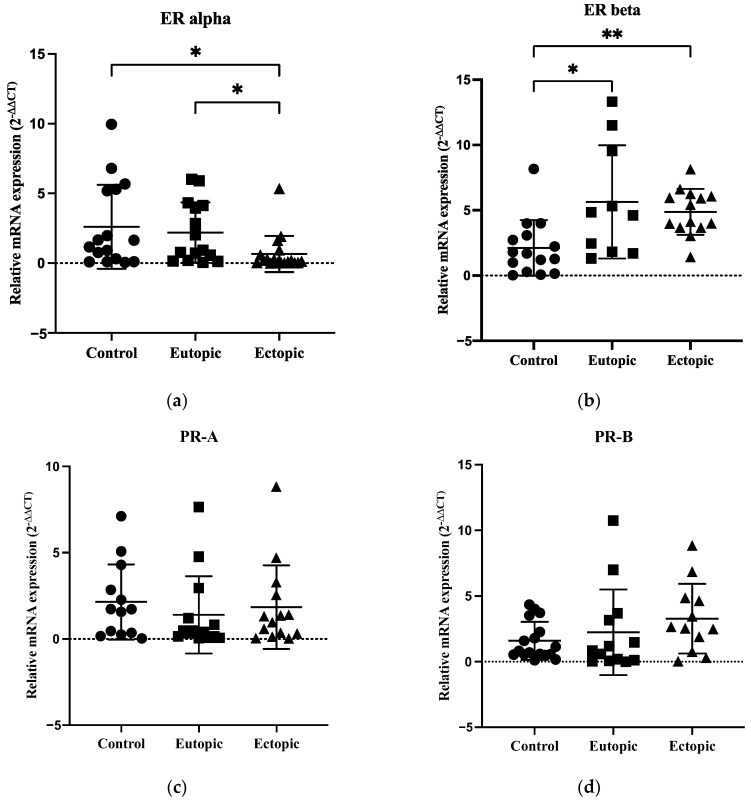
qRT-PCR analysis of ER and PR expressions in controls, eutopic and ectopic groups. (**a**) ERα expression was significantly decreased, (**b**) ERβ expression was significantly increased, whereas (**c**,**d**) PR-A and PR-B expression did not show significant differences between the groups. Data are presented as mean ± SD and analysed using Kruskal–Wallis with Dunn’s multiple comparisons test. * *p* < 0.05 and ** *p* < 0.01.

To determine the presence of progesterone resistance in women with endometriosis, qRT-PCR was performed to determine the mRNA levels of ERα, ERβ, PR-A and PR-B in the samples. The expression of ERα was significantly downregulated in the ectopic group compared to the eutopic and control groups (*p* = 0.0215 and *p* = 0.0186 respectively) (Figure 4a). However, the expression of ERβ was significantly upregulated in the eutopic and ectopic groups compared to the control group (*p* = 0.0257 and *p* = 0.0039, respectively) (Figure 4b). PR-A and PR-B expression showed no significant differences between the groups (Figure 4c,d). The raw data for ER and PR expression is shown in Supplementary Table S6.

### 3.6. Target Prediction of Hsa-miR-199a-3p, Hsa-miR-1-3p and Hsa-miR-125b-5p

The target genes of hsa-miR-199a-3p, hsa-miR-1-3p and hsa-miR-125b-5p were predicted by StarBase, which consists of an interaction map between miRNA and mRNA. It was found that hsa-miR-199a-3p was upregulated with reduced expression of the target genes SCD, TAOK1 and DDIT4. The probability that hsa-miR-199a-3p preferentially conserved the SCD, TAOK1 and DDIT4 genes was 0.86, 0.96 and 0.78, respectively. In addition, hsa-miR-1-3p was upregulated upon reduced expression of LASP1, CDK6, TAGLN2 and G6PD target genes. The P_CT_ of LASP1, CDK6, TAGLN2 and G6PD by hsa-miR-1-3p was 0.7, 0.85, 0.89 and >0.99, respectively. Finally, hsa-miR-125b-5p was upregulated, and the ELOVL6 target gene was downregulated with a P_CT_ of 0.92 (Table 4). 

## 4. Discussion

Patients with endometriosis are presented with infertility, dysmenorrhoea, dyspareunia and pelvic pain [3]. Although progestin therapy provides temporary relief of pelvic pain, patients still respond poorly to treatment due to progesterone resistance [8,9,10]. One of the causes of progesterone resistance could be the epigenetic regulation of gene expression by miRNAs [9,17]. Previous studies have shown that the aberrant expression of ER, PR, miR-29c, miR-135a, miR-135b, miR-194-3p, miR-196a and miR-92a may contribute to progesterone resistance and impaired decidualisation in endometriosis [22,23,24,25,41,42,43,44,45,46,47,48,49,50,51]. 

Our NGS study detected three significantly upregulated miRNAs (miR-1247-3p, miR-1246 and novel-miR-243-5p) and four significantly downregulated miRNAs (miR-1973, miR-199a-3p, miR-181a-2-3p and miR-7-5p) in ectopic endometriosis samples. To the best of our knowledge, this is the first study to discover significant results for miR-1247-3p, miR-1246, novel-miR-243-5p, miR-1973 and miR-181a-2-3p in endometriosis patients. These miRNAs were also found to be dysregulated in other diseases. Previous studies reported an increase in miR-1247-3p in tumour-derived exosomes, promoting lung metastasis in hepatocellular carcinoma and angiogenesis in bladder carcinoma [52,53], which is consistent with our result on the upregulation of miR-1247-3p. Moreover, the overexpression of miR-1246 is consistent with studies that demonstrate the oncogenic role of miR-1246 in the progression of various cancers, such as colorectal, breast, renal and ovarian cancers [54]. MiR-243-5p is the first novel gene we have found in humans that is also present in *Caenorhabditis elegans*, namely cel-miR-243-5p. The sequence of cel-miR-243-5p is 20-UAUCUCGGUGCGAUCGUAC–38 [55]. The downregulation of miR-1973 is consistent with previous studies showing reduced miR-1973 expression in patients with sperm abnormalities and renal cell carcinoma [56,57]. The reduced expression of miR-181a-2-3p in our NGS data is consistent with Liang et al. (2022) [58] who also demonstrated downregulation of miR-181a-2-3p in myelodysplastic syndrome, but not with Li et al. (2021), who showed upregulation of miR-181a-2-3p and downregulation of the MYLK target gene in gastric cancer [59]. The downregulation in miR-7-5p in our study is in line with Antonio et al. (2023), who showed a significant reduction of miR-7-5p expression in superficial peritoneal endometriosis compared to deep infiltrating endometriosis and ovarian endometrioma [60]. The NGS investigation in our study detected a downregulation of miR-199a-3p in ectopic endometriosis samples, which is consistent with previous studies showing a reduction of miR-199a-3p in human endometriotic cyst stromal cells (ECSCs) [61] and in the plasma of endometriosis patients [62]. However, it is inconsistent with our qRT-PCR validation result, which showed an upregulation of miR-199a-3p in the ectopic group compared to the eutopic group, and Walasik et al. (2023) who did not detect significant differences in miR-199a-3p expression between endometriosis and control groups in plasma samples [63].

However, only miRNAs that are associated with the role of endometriosis, such as miR-199a-3p [61], miR-196a-5p [25], miR-144-3p [64], miR-1-3p [65], miR-146a-5p [66] and miR-125b-5p [67] were selected from volcano plots and heatmaps for validation by qRT-PCR (Figure 1e). MiR-199a-3p plays an important role in cell invasion, motility and contractility and as a diagnostic biomarker in endometriosis [61,62]. MiR-196a may contribute to progesterone resistance in endometriosis [25] and miR-144-3p has been shown to correlate with cell survival status in human endometriotic lesions and is involved in the regulation of inflammatory mediators such as IL-6, IL-1β, TNFα, PTGS2 and COX2 [64]. Furthermore, miR-1-3p could be a tumour suppressor gene to differentiate between endometriosis and ovarian cancer while monitoring the risk of malignant transformation from endometriosis to ovarian cancer [65,68]. MiR-146a-5p may play a significant role in angiogenesis and infertility due to decreased endometrial receptivity in endometriosis [66,69,70], whereas miR-125b-5p could be a good diagnostic biomarker in endometriosis [67,71,72].

In our qRT-PCR validation data, there were no significant differences in miR-196a-5p expression between the three groups, which contrasts with a previous study showing an upregulation of miR-196a in the eutopic endometrium of patients with endometriosis [25]. There was a significant downregulation of miR-144-3p expression between eutopic and control groups, but no significant differences were observed between ectopic and eutopic samples in endometriosis patients. The result is not consistent with a previous study showing a significant overexpression of miR-144-3p in ectopic versus eutopic tissue in endometriosis [64]. Furthermore, the expression of miR-1-3p was significantly upregulated in ectopic compared with eutopic and control samples, which is consistent with previous studies showing upregulation of miR-1-3p in endometriotic lesions compared to ovarian cancer and control groups [65,68]. The expression of miR-146a-5p was significantly upregulated in ectopic compared to eutopic samples but significantly downregulated in eutopic versus control groups in patients with endometriosis. This result is consistent with Ji et al. (2024) [73] who demonstrated a significant upregulation of miR-146a-5p in ectopic endometrial stromal cells compared with eutopic tissues and exosomes but is not consistent with previous studies showing significant downregulation of miR-146a-5p in endometriotic tissues compared to the control group [66] and significant upregulation of miR-146a-5p in eutopic endometrium of patients with endometriosis compared with the control group [69,70]. Finally, the expression of miR-125b-5p was significantly upregulated in ectopic endometriotic tissues compared to the eutopic group. This result is in line with Moustafa et al. (2020) [67] and Cosar et al. (2016) [71], who found a significant upregulation of miR-125-5p in the serum of patients with endometriosis. However, Walasik et al. (2023) were unable to detect any significant differences in miR-125b-5p expression between endometriosis and control groups in plasma samples [63].

There were discrepancies between the results of NGS and qRT-PCR validation data, in which miR-199a-3p was significantly downregulated when ectopic and control groups were compared in NGS but significantly upregulated in the ectopic versus the eutopic group in qRT-PCR. In addition, no significant differences in the expression of miR-196a-5p, miR-144-3p, miR-1-3p, miR-146a-5p and miR-125b-5p were detected between the groups by NGS. However, qRT-PCR validation revealed a significant upregulation of miR-1-3p, miR-146a-5p and miR-125b-5p in the ectopic compared to the eutopic group, while the expression of miR-144-3p was significantly downregulated in the eutopic samples compared to the control group. The differences in the results could be due to the possible higher sensitivity of the qRT-PCR technique compared to high throughput technologies such as NGS and microarray [74,75]. Moreover, miRNAs with different functions and molecular pathways are differentially expressed between ectopic and eutopic groups in endometriosis [20,76]. In our study, qRT-PCR validations of miR-199a-3p, miR-1-3p, miR-146a-5p and miR-125b-5p showed reduced expression in the eutopic group compared to the ectopic group. This could be due to the small amount of tissue collection during pipelle sampling [77] and possible retrograde menstruation, in which endometrial tissue from the uterine lining enters the ectopic lesions [4,5]. The variations in miRNA expression in both NGS and qRT-PCR could be due to the heterogeneity of endometriotic lesions that consist of a mixture of endometrium and other types of tissues, masking the changes in miRNA expressions [78]. In addition, the inconsistency of the results could be due to exosomes secreted from the endometrium and uterus into the bloodstream [79]. Studies have shown that exosomal miR-6795-5-3p, miR-22-3p and miR-320a biomarkers were upregulated in the serum of patients with endometriosis [80,81].

The imbalances between the expression of ER and PR could contribute to the pathophysiology of endometriosis [13]. In our study, the expression of ERα was significantly downregulated, and the expression of ERβ was significantly upregulated in endometriosis compared to the control group. This result is consistent with previous studies showing significantly decreased expression of ERα and increased expression of ERβ in endometriosis [46,47,48,49]. However, Matsuzaki et al. (2001) showed increased expression of ERα and reduced expression of ERβ in endometriotic tissue [50]. ERβ is known to be a key mediator of inflammation induced by high estradiol levels and could suppress the expression of ERα in endometriosis [13,46]. We also observed that PR-A and PR-B expression showed no significant differences between the groups. Our results were not consistent with previous studies that had shown downregulation of PR [24,25,26,82], increased PR-A and decreased PR-B expression in endometriosis [41,42,43,44]. It is known that aberrant PR expression plays a role in progesterone resistance and impaired decidualisation in endometriosis [24,25,26,82]. However, ER and PR are not direct targets of the upregulated miRNAs, such as miR-199a-3p, miR-1-3p, miR-146a-5p and miR-125b-5p. The variations of ER and PR expressions in endometriotic lesions might be due to the heterogeneity of hormone receptors in the same section of endometriotic tissue [83,84]. 

**Figure 5:**
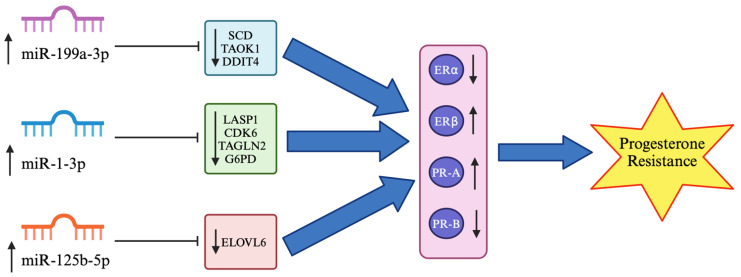
Regulation of miR-199a-3p, miR-1-3p and miR-125b-5p on target genes and steroid hormone receptors in endometriosis. The model is based on the results of the current study and literature data. MiR-199a-3p has been shown to bind directly and downregulate SCD, TAOK1 and DDIT4 expressions [32,33,34,35]. In addition, miR-1-3p overexpression could target LASP1, CDK6, TAGLN2 and G6PD mRNA [36,37,38,39], whereas miR-125b-5p upregulation could reduce ELOVL6 expression in endometriosis [40]. These three miRNAs could indirectly regulate ER and PR expression by increasing ERβ and decreasing ERα expression while upregulating PR-A and downregulating PR-B expression [41,42,43,44], thus contributing to progesterone resistance in endometriosis. ↑ indicates upregulation, ↓ indicates downregulation, ⊣ indicates miRNA inhibition on target genes whereas ⇨ indicates the next process.

Our data on the upregulation of miR-199a-3p, miR-1-3p, miR-146a-5p and miR-125b-5p with significantly increased ERβ and reduced ERα but no significant differences in PR-A and PR-B expression partially support our hypothesis. Three miRNAs (miR-199a-3p, miR-1-3p and miR-125b-5p) may bind directly to the SCD, TAOK1, DDIT4, LASP1, CDK6, TAGLN2, G6PD and ELOVL6 target genes, which may indirectly regulate ER and PR expression, contributing to progesterone resistance in endometriosis (Table 4 and Figure 5). The inhibitors of these three miRNAs that lead to upregulation of the target genes could be potential therapeutic targets in endometriosis. However, the upregulation of these miRNAs and the downregulation of the target genes could be used as targeted therapies for specific diseases. Upregulation of miR-199a-3p could reduce adipocyte differentiation by targeting SCD, which could alter the composition of fat in the body and reduce the risk of obesity [32]. The overexpression of MiR-199a-3p could also stimulate cardiomyocyte proliferation and cardiac regeneration after myocardial infarction by binding to TAOK1 and activating YAP [34]. In addition, increased expression of miR-199a-3p downregulates the apoptotic target gene DDIT4, which is cardioprotective and enhances the therapeutic effect of carvedilol in ischaemia/reperfusion injury [35]. Prostate cancer cells transfected with an miR-1-3p mimic downregulate the expression of the target gene LASP1, which reduces cell viability, invasion, and migration in prostate cancer [36]. Transfection of human colon carcinoma cells with an miR-1-3p mimic directly targeting CDK6 could increase apoptosis and cell cycle arrest in colon carcinomas [37]. Quercetin therapy could reduce growth and invasion but increase cell apoptosis while upregulating miR-1-3p expression and downregulating TAGLN2 expression in oesophageal cancer [38]. Furthermore, an increase in miR-1-3p expression leads to the downregulation of the G6PD target gene with a decrease in cell proliferation and aerobic glycolysis but increases cell apoptosis in gastric cancer cells. Aerobic glycolysis enhances tumour metastasis by increasing the uptake of glucose and lactate and ATP production [39]. Finally, miR-125b-5p has been shown to bind directly with the ELOVL6 target gene and reduce its expression. Patients with glioblastoma multiforme and hepatocellular carcinoma have poor prognosis due to high ELOVL6 levels. Therefore, the downregulation of ELOVL6 by increasing miR-125b-5p expression could be used as a targeted therapy to treat these diseases [40].

One of the limitations of this study is the lower number and heterogeneity of samples in each group, which may partly explain the contradiction between screening and validation data. In the qRT-PCR validation data, the heterogeneity of samples leads to high variations in miRNA and gene expression, which could contribute to insignificant results in miR-196a-5p, PR-A and PR-B expression. In addition, when comparing three groups of patients (control, eutopic and ectopic groups), it is more difficult to obtain significant results between the groups after statistical analysis [74]. The diagnosis between the control and endometriosis groups may be challenging as the histological diagnosis is the same in the three groups. Moreover, the clinical features of the control group may be similar to those of the endometriosis group. Therefore, invasive surgery by laparoscopy is required to confirm the diagnosis by visualising all signs of an endometriotic lesion.

In future studies, we would increase the sample size to improve statistical power and obtain more accurate estimates and more meaningful differences to answer the research questions. This can be performed by calculating the sample size and performing power analysis using the G*Power software (version 3.1.9.7) [85,86]. The expression of the miRNAs could be determined by different phases of the menstrual cycle, such as proliferative, early secretory, mid secretory and late secretory, or according to the stages of endometriosis (I-IV). In addition, analysing tissue samples of surgically induced mouse models with endometriosis at 3 days, 2 weeks, 4 weeks and 8 weeks post-surgery could provide a more dynamic view of the temporal changes of miRNAs, ER and PR in endometriosis [83,87]. For functional validation experiments, transfection of endometriotic cell lines with miRNA mimics or inhibitors could be performed to determine the effects of upregulation or downregulation of miRNA expression on target genes and cell behaviours. The expression of predicted target genes could be measured by qRT-PCR, and the binding of miRNA to the 3’UTR of target genes can be confirmed by luciferase reporter assay. Cell behaviour can also be studied by performing cell proliferation, invasion and apoptosis assays [28]. In vivo studies on mice can be carried out by autotransplanting the endometrium from one side of the uterine horn into the peritoneal cavity. Then, the mice could be divided into two groups and injected intraperitoneally with a miRNA inhibitor or an inhibitor control for miR-199a-3p, miR-1-3p, miR-146a-5p or miR-125b-5p. One month later, the mice could be sacrificed, and the lesion harvested to measure the expression levels of miRNA and target genes for therapeutic efficacy. To assess therapeutic efficacy, a randomised controlled trial could also be performed, in which subjects are randomly assigned to two groups, with one group of patients receiving miRNA inhibitor treatment and the other group receiving a placebo as a control. Blood samples could be taken before and after 2 months of treatment to measure the miRNA and target gene expression. Furthermore, detailed analyses of signalling pathways can be performed using Reactome, Gene Ontology, Kyoto Encyclopaedia of Genes and Genomes (KEGG) and STRING databases [88]. The identified pathways could be validated by Western blot using primary antibodies. After incubation with secondary antibodies, the protein expressions could be viewed as bands with enhanced chemiluminescence and measured using Image J software (version 1.54k). Due to the high variability of ER and PR gene expression, ER and PR protein expressions could be measured by western blot for validation in the future. Other hormone receptors or signalling molecules that could be investigated are chicken ovalbumin upstream promoter-transcription factor II (COUP-TFII), heart and neural crest derivatives expressed 2 (HAND2) and steroid receptor coactivator-1 (SRC-1), which are related to progesterone and estrogen signalling in endometriosis [89].

## 5. Conclusions

In conclusion, we found that the expression of miR-199a-3p, hsa-miR-1-3p, hsa-miR-146a-5p, hsa-miR-125b-5p and ERβ was significantly upregulated, while the expression of ERα was significantly downregulated in ectopic endometriotic tissues. However, PR-A and PR-B expression showed no significant differences between the groups. To our knowledge, this is the first study to determine the roles of miR-199a-3p, hsa-miR-1-3p and hsa-miR-125b-5p in progesterone resistance in endometriosis via targeting SCD, TAOK1, DDIT4, LASP1, CDK6, TAGLN2, G6PD and ELOVL. Therefore, this research contributes to a better understanding of the miRNA expression profile, steroid hormone receptor expression and the target genes involved in endometriosis. This offers exciting opportunities for the development of biomarkers and personalised, targeted therapies for this debilitating disease.

## Figures and Tables

**Table 1:** Patient demographic data of samples used in the study.

Characteristics	Control Samples N (%)	Eutopic Samples N (%)	Ectopic Samples N (%)
Mean age ± S.D. (years)	36.94 ± 7.04	35.28 ± 6.52	37.50 ± 7.29
Race			
Malay	12 (66.67%)	16 (88.89%)	16 (88.89%)
Non-Malay	6 (33.33%)	2 (11.11%)	2 (11.11%)
Parity			
Nulliparous	10 (55.56%)	13 (72.22%)	12 (66.67%)
Multiparous	8 (44.44%)	5 (27.78%)	6 (33.33%)
Mean BMI ± S.D. (kg/m^2^)	25.65 ± 3.38	24.74 ± 3.39	26.28 ± 5.55
Clinical Features			
Infertility	7 (38.89%)	11 (61.11%)	9 (50%)
Dysmenorrhoea *	10 (55.56%)	14 (77.78%)	17 (94.44%)
Dyspareunia	3 (16.67%)	6 (33.33%)	6 (33.33%)
Pelvic Pain	5 (27.78%)	8 (44.44%)	11 (61.11%)

* There is a significant difference in dysmenorrhoea between ectopic and control samples (*p* = 0.024).

**Table 2:** MiRNAs significantly upregulated and downregulated in ectopic endometriosis compared to control group.

miRNAs	Adjusted *p*-Value	Log2 Fold Change
Upregulated		
hsa-miR-1247-3p	0.0091	2.59
Downregulated		
hsa-miR-1973	8.63 × 10^−6^	−22.99
hsa-miR-199a-3p	7.93 × 10^−6^	−7.16
hsa-miR-181a-2-3p	0.048	−1.55

**Table 3:** MiRNAs significantly upregulated and downregulated in ectopic versus eutopic group in endometriosis patients.

miRNAs	Adjusted *p*-Value	Log2 Fold Change
Upregulated		
hsa-miR-1246	0.0052	5.45
novel-hsa-miR-243-5p	0.041	3.49
Downregulated		
hsa-miR-7-5p	0.0052	−7.57

**Table 4:** Expression levels of miRNAs and target genes identified in StarBase with probability of preferentially conserved targeting (P_CT_).

Expression Level of miRNA	Expression Level of Target Gene	Tissue or Cell Type	P_CT_	References
Upregulated: hsa-miR-199a-3p	Reduced SCD	Adipocyte, ovine mammary epithelial cells	0.86	Tan et al. (2017) [32], Wang et al. (2022) [33]
	Reduced TAOK1	Cardiomyocyte	0.96	Torrini et al. (2019) [34]
	Reduced DDIT4	Cardiomyocyte	0.78	Park et al. (2016) [35]
Upregulated: hsa-miR-1-3p	Reduced LASP1	Prostate cancer	0.7	Guo et al. (2023) [36]
	Reduced CDK6	Human colon carcinoma cells	0.85	Fragoso et al. (2022) [37]
	Reduced TAGLN2	Esophagus carcinoma cells	0.89	Wang et al. (2022) [38]
	Reduced G6PD	Gastric cancer cell	>0.99	Deng et al. (2021) [39]
Upregulated: hsa-miR-125b-5p	Reduced ELOVL6	HEK-293 cells	0.92	Istiqamah et al. (2023) [40]

## Data Availability

Restrictions apply to the availability of these data. Data were obtained from BGI and are available at https://biosys.bgi.com/ (accessed on 7 April 2022) with the permission of BGI.

## Supplementary Materials

The following supporting information can be downloaded at https://www.mdpi.com/article/10.3390/biomedicines12102218/s1, Supplementary Figure S1: H&E staining of tissues from patients with and without endometriosis. Supplementary Table S1: Total number of reads in NGS, Supplementary Table S2: Reads alignment with reference genome; Supplementary Table S3: Small RNA classification; Supplementary Table S4: Gene expression TPM and CPM; Supplementary Table S5: miRNA validation qPCR raw data; Supplementary Table S6: mRNA qPCR raw data.
