# Supplementary material for: Exploring the Role of MicroRNAs in Progesterone and Estrogen Receptor Expression in Endometriosis
Source: Biomedicines. 2024 Sep 28;12(10):2218. doi: 10.3390/biomedicines12102218 (PMC11504708; doi:10.3390/biomedicines12102218)
Supplement: Supplementary file 1 [file biomedicines-12-02218-s001.zip › Supplementary Figure S1 H&E Staining.pdf]

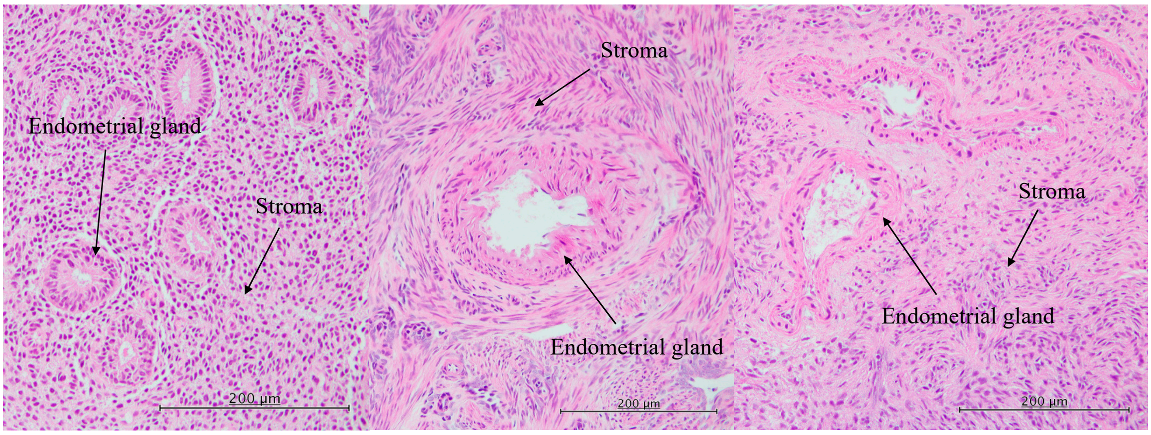

Normal endometrium Eutopic endometrium Ectopic endometriotic tissue

Figure S1. H&E staining of tissues from patients with and without endometriosis. Microscopic examination showed structures of endometrial glands and stroma in normal endometrium (n=18), eutopic endometrium (n=18), and ectopic endometriotic tissue (n=18) from ovarian cyst. (Magnification x200).
